# Nutritional intervention with cyanidin hinders the progression of muscular dystrophy

**DOI:** 10.1038/s41419-020-2332-4

**Published:** 2020-02-18

**Authors:** Marielle Saclier, Chiara Bonfanti, Stefania Antonini, Giuseppe Angelini, Giada Mura, Federica Zanaglio, Valentina Taglietti, Vanina Romanello, Marco Sandri, Chiara Tonelli, Katia Petroni, Marco Cassano, Graziella Messina

**Affiliations:** 1 Department of Biosciences, University of Milan, via Celoria 26, 20133 Milan, Italy; 2 Venetian Institute of Molecular Medicine (VIMM), Department of Biomedical Sciences, University of Padova, Padova, Italy

**Keywords:** Mechanisms of disease, Skeletal muscle

## Abstract

Muscular Dystrophies are severe genetic diseases due to mutations in structural genes, characterized by progressive muscle wasting that compromises patients’ mobility and respiratory functions. Literature underlined oxidative stress and inflammation as key drivers of these pathologies. Interestingly among different myofiber classes, type I fibers display a milder dystrophic phenotype showing increased oxidative metabolism. This work shows the benefits of a cyanidin-enriched diet, that promotes muscle fiber-type switch and reduced inflammation in dystrophic *alpha-sarcoglyan* (*Sgca)* null mice having, as a net outcome, morphological and functional rescue. Notably, this benefit is achieved also when the diet is administered in dystrophic animals when the signs of the disease are seriously evident. Our work provides compelling evidence that a cyanidin-rich diet strongly delays the progression of muscular dystrophies, paving the way for a combinatorial approach where nutritional-based reduction of muscle inflammation and oxidative stress facilitate the successful perspectives of definitive treatments.

## Introduction

Muscular dystrophies (MDs) are a group of heterogeneous genetic diseases, characterized by wasting of skeletal muscle tissue, which over time compromises patient mobility and, in the most severe cases, respiratory and cardiac functionality leading to premature death^1,2^. In many cases, the mutations affect one or more proteins that cluster in the dystrophin-glycoprotein complex (DGC) located on the sarcolemma of the myofibers and links it to the basal lamina. This complex connects myofibers to the extracellular matrix, and its role is essential for fiber integrity and cell signaling during contraction. The mutations result in the disassembly and/or a malfunction of the entire DGC, which leads to increased fragility of sarcolemma and myofibers death. Damaged and dead fibers can be replaced by satellite cells (SCs), the adult stem cells of skeletal muscle tissue. In this pathologic scenario, since SCs share the same mutation as well as the damaged myofibers, they differentiate in fragile myofibers, leading to a loop of degeneration and regeneration^2^. In time, the population of SCs is exhausted and the damaged muscle is replaced by connective and adipose tissue, impairing the physiological function of muscle tissue^1–3^. Despite the molecular mechanisms behind MDs are partially known, this class of diseases is one of the most difficult to treat. Indeed, although several clinical trials have been carried on, MDs are still orphan diseases^4^.

Skeletal muscle is the most abundant tissue in the human body and it is composed of large multinucleated fibers, whose nuclei cannot divide. Consequently, any cell or gene replacement strategy must restore proper gene expression in hundreds of millions of post-mitotic nuclei, which are embedded in a highly structured cytoplasm and surrounded by a thick basal lamina. It is therefore evident that, although caused by a single gene defect, this group of pathologies could be considered as multifactorial: misregulation of associated sarcoplasmic proteins, severe chronic inflammation and consequent macrophage infiltration resulting in fibrosis. Among the different approaches, many efforts are directed to slow down the progression of the disease to counteract progressive degeneration and to improve patients’ quality life^5^. Several pieces of evidence showed that oxidative stress and accumulation of reactive oxygen species (ROS) strongly contribute to aggravate the dystrophic pathology^6,7^. One of the considered strategies is to use antioxidant molecules to counteract the oxidative stress generated by muscle contraction and degeneration^6–13^.

Another important aspect of MD progression is chronic inflammation, as the secretion of several cytokines recalls macrophages, essential players in acute muscle regeneration. However, in a chronic myopathic context, this population establishes a sustained inflammatory milieu worsening the dystrophic phenotype^14,15^.

Anthocyanins are a subclass of flavonoids found in pigmented plants, widely recognized for their anti-inflammatory and antioxidant properties^16,17^. Preventive effects of dietary anthocyanins have been described in epidemiological and preclinical studies, indicating health-promoting properties against cardiovascular disease, cancer, and neurodegenerative diseases^16,18^. More than 700 different anthocyanins have been reported in nature, each identified by specific glycosylation, methylation, and acylation of the aglycones anthocyanidins (i.e., cyanidin, delphinidin, malvidin, pelargonidin, peonidin, and petunidin)^17^. Anthocyanin-rich corn mainly contains cyanidin 3-glucoside and its acylated derivatives^19^. Dietary intake of cyanidin 3-glucoside from anthocyanin-rich corn reduced myocardial injury upon ischemia-reperfusion and against cardiotoxic effects induced by Doxorubicin, an anthracycline widely used as chemotherapeutic drug against a variety of cancer types^20,21^.

In this study, we produce evidence that dietary intake of cyanidin 3-glucoside (here referred to as cyanidin), an anthocyanin from purple corn^21^, is beneficial for treating MD pathologies. We indeed provide significant results that cyanidin-enriched diet (Red diet, RD) supplied to the dystrophic *alpha-sarcoglycan* null mouse model^22^ either at weaning or adulthood, when the signs of the disease are already present, improves the morphological and functional recovery of the pathologic phenotype. Additionally, we suggest that cyanidin supplementation promotes mitochondrial biogenesis which in turn preserves muscle function.

## Results

### Cyanidin-enriched diet ameliorates the histopathological condition of dystrophic muscles

To establish whether cyanidin supplementation could affect the onset and progression of dystrophic conditions, we fed *Sgca* null mice at weaning with a cyanidin-enriched diet either for 5 or 25 weeks, to evaluate, respectively, the short and long-term nutritional benefits. As a control, we also provide *Sgca* null and wild-type mice (WT) with a cyanidin-free diet isogenic to RD, referred to as Yellow diet (YD) (Fig. S1a). The *Sgca* null mouse model^22^ was chosen considering its severity that makes it resemble the human Duchenne Muscular Dystrophy (DMD) pathology with respect to the usually studied *mdx* mouse.

**Fig. 1:**
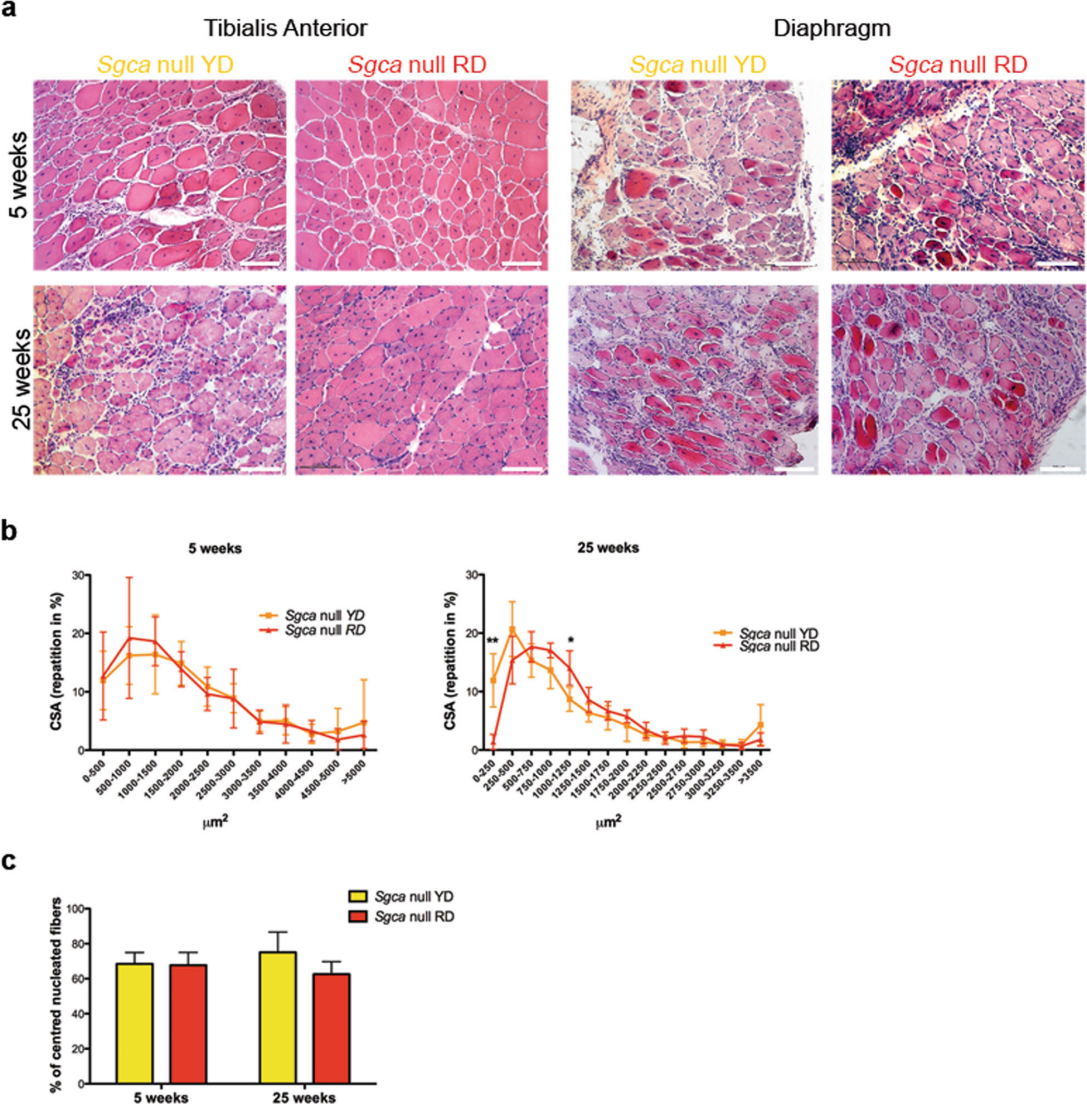
Cyanidin-enriched diet ameliorates muscle morphology. **a** Hematoxylin and Eosin (H&E) staining of Tibialis Anterior and Diaphragm of *Sgca* null mice fed with YD or RD for 5 or 25 weeks. Scale bar 100 μm. For Tibialis Anterior: *N* = 6 *Sgca* null YD and RD mice in the 5-week group, *N* = 8 *Sgca* null YD mice and *N* = 10 *Sgca* null mice RD mice for the 25-week group. For Diaphragm: *N* = 6 *Sgca* null YD and RD mice in the 5-week group, *N* = 8 *Sgca* null YD mice and *N* = 10 *Sgca* null mice RD for the 25-week group. **b** Distribution of myofibers Cross Sectional Area (CSA) of Tibialis Anterior of *Sgca* null mice fed with YD or RD for 5 or 25 weeks. *N* = 6 for *Sgca* null YD mice, *N* = 7 for *Sgca* null RD mice in the 5-week group. *N* = 4 for *Sgca* null YD and RD mice in the 25-week group. Results are means ± SD. Two-tailed unpaired Student’s *t*-test; *P < 0.05; **P < 0.01. **c** Percentage of centered nucleated fibers in Tibialis Anterior sections of *Sgca* null mice fed for 5 and 25 weeks with YD or RD. *N* = 8 *Sgca* null YD and RD mice for the 5 and 25-week groups. Results are means ± SD.

First, to evaluate whether the two diets do not influence the normal growth and development of the mouse model, we analyzed the food income and the body weight for 5-week post weaning in *Sgca* null mice. Compared to *Sgca* null mice fed with a standard diet, we did not observe differences in both parameters (Fig. S1b and not shown). Then, we analyzed muscle histology through Hematoxylin and Eosin staining on *Tibialis anterior* (TA) and diaphragm sections. While dystrophic mice fed with control YD for 5-week-display the first signs of muscle degeneration with inflammatory infiltrates and necrotic areas that progressively degenerate, RD-fed *Sgca* null mice appeared with an improved morphology of muscle tissue, with less infiltrates yet at 5-week-time point (Fig. 1a, top panels). At 25-week-time point, RD-fed *Sgca* null mice show preserved muscle morphology with reduced necrosis and cellular infiltrates when compared with YD-fed counterpart (Fig. 1a, bottom panels). Consistently, the measurement of fiber size distribution (Cross-Sectional Area, CSA) in TA sections confirmed the cyanidin-induced morphological ameliorations at 25 weeks in RD-fed dystrophic mice with increased calibre homogeneity compared with the YD-fed counterpart (Fig. 1b). The analysis of centrally nucleated myofibers, as an index of muscle regeneration, did not show any significant difference between YD- and RD-fed animals, indicating that cyanidin supplementation does not affect the regeneration process (Fig. 1c).

**Fig. 2:**
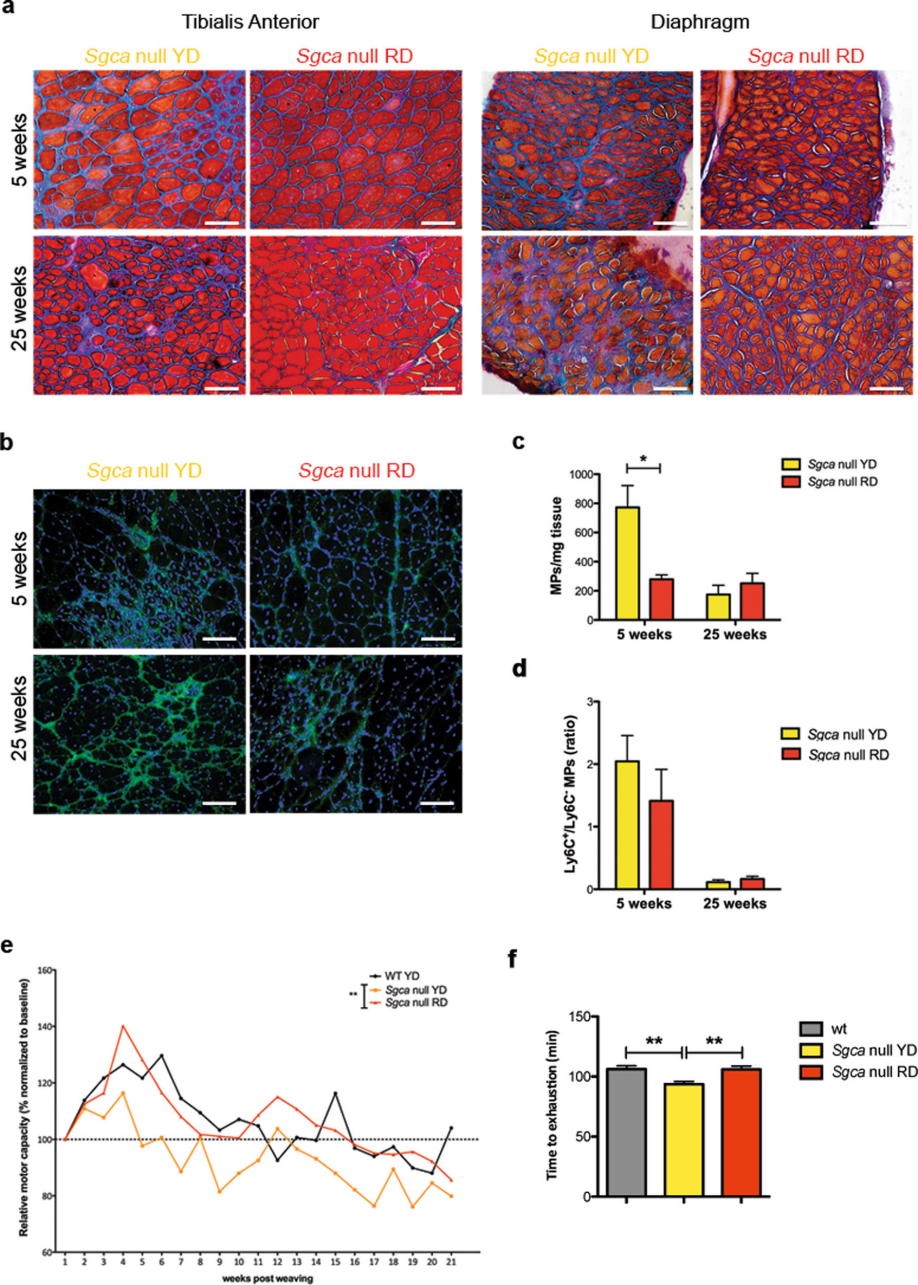
Cyanidin-enriched diet decreases collagen I deposition, macrophages infiltration and rescue muscle performance. **a** Milligan’s Trichrome staining of Tibialis Anterior and Diaphragm of *Sgca* null mice fed with YD or RD for 5 or 25 weeks. Scale bar 100 μm. For Tibialis Anterior: *N* = 6 *Sgca* null YD and RD mice in the 5-week group, *N* = 8 *Sgca* null YD mice and *N* = 10 *Sgca* null RD mice for the 25-week group. For Diaphragm: *N* = 6 *Sgca* null YD and RD mice in the 5-week group, *N* = 8 *Sgca* null YD mice and *N* = 10 *Sgca* null RD mice for the 25-week group. **b** Collagen I Immunofluorescence (green) and nuclei (blue) in Tibialis Anterior sections of *Sgca* null mice fed for 5 and 25 weeks with YD or RD. Scale bar 100 μm. *N* = 4 *Sgca* null YD and RD mice in the 5-week group. *N* = 5 *Sgca* null YD and RD mice in the 25-week group. **c** Number of MPs from total hind limb lysates of *Sgca* null mice fed with YD or RD for 5 or 25 weeks. Results are means ± SD; Two-tailed unpaired Student’s *t*-test; *P < 0.05. *N* = 6 for *Sgca* null YD mice and *N* = 5 for *Sgca* null RD mice in 5-week group. *N* = 4 for *Sgca* null YD mice and *N* = 3 for *Sgca* null RD mice in 25-week group. **d** Ratio between LyC6^+^ and LyC6^-^ MPs from total hind limb lysate of *Sgca* null mice fed with YD or RD for 5 and 25 weeks. *N* = 6 for *Sgca* null YD mice and *N* = 5 for *Sgca* null RD mice in 5-week group. *N* = 4 for *Sgca* null YD mice and *N* = 3 for *Sgca* null RD mice in 25-week group. Results are means ± SD; Two-tailed unpaired Student’s *t*-test. **e**, **f** Treadmill test (time to exhaustion) over time and **f** the total average of the measurements of WT mice fed with YD and *Sgca* null mice fed with YD or RD. *N* = 3 WT mice, *N* = 4 *Sgca* null YD and RD mice. Results are means ± SD; Two-tailed unpaired Student’s *t*-test; **P < 0.01.

Collagen deposits are hallmarks of myopathy in MDs and compromise patients’ mobility by replacing muscle tissue upon chronic inflammatory cues^23^. To establish whether cyanidin-dietary enrichment might impact on collagen deposits, we performed Milligan’s trichrome staining on TA and diaphragm of YD- and RD-fed *Sgca* null animals. As shown in Fig. 2a, while YD-fed dystrophic muscles accumulate abundant extracellular matrix deposits particularly at 25 weeks, the TA and diaphragm muscles of RD-fed animals exhibit a prolonged reduction of extracellular matrix deposition at both time points analyzed.

To strengthen these observations, we quantified the fluorescent positive area of TA sections stained for collagen I (Fig. 2b) revealing a drastic reduction of its deposition in RD- vs. YD-fed dystrophic mice both at 5 and 25 weeks from the diet regimen (Fig. S1c).

Inflammation accelerates the clinical progression of MDs and this is mainly accounted for by muscle-infiltrating macrophages that engender a chronic inflammatory milieu^24^. To assess the macrophage infiltration status, we FAC-sorted CD64^+^ cells from hind-limb muscle lysates of *Sgca* null mice fed with either YD or RD. As shown in Fig. 2c, the total macrophage population significantly drop in the muscle of dystrophic mice following cyanidin dietary enrichment. However, the ratio of pro-inflammatory to anti-inflammatory macrophage sub-populations (CD64^+^-Ly6C^+^ and CD64^+^-Ly6C^−^, respectively) was not significantly affected by the diet at both time points (Fig. 2d). These observations suggest that cyanidin may facilitate an anti-inflammatory effect within the muscle environment, which is independent by macrophage phenotype.

Progressive reduction of muscle performance mainly characterizes MDs, thus we sought to investigate whether the cyanidin-enriched diet may beneficially impact this condition in *Sgca* null mice with the treadmill test. As an index of performance, animals after 5 weeks supplied with RD or YD were measured in terms of time to exhaustion over the following 15 weeks maintaining the dietary protocol. Cyanidin supplementation was sufficient to promote significant and prolonged muscle endurance of dystrophic animals compared to their control YD-fed littermates (Fig. 2e, f). Notably, the performance of *Sgca* null mice fed with the RD was comparable to the WT group.

### The cyanidin-enriched diet promotes a shift towards an oxidative metabolism

**Fig. 3:**
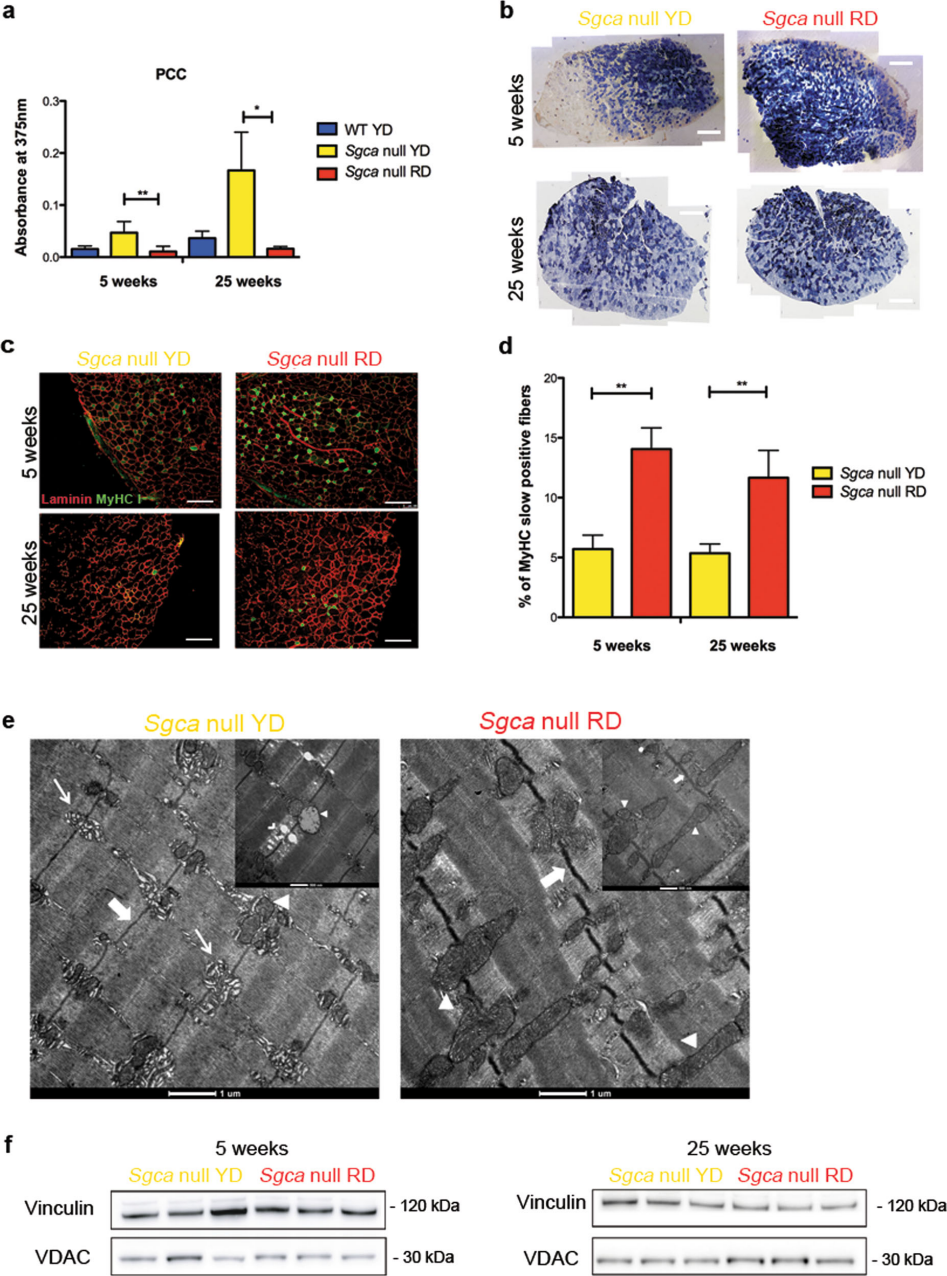
Cyanidin-enriched diet promotes a shift to a more oxidative fiber metabolism. **a** Quantification of protein carbonylation content (PCC) from protein lysate of WT, *Sgca* null YD or RD mice fed for 5 or 25 weeks. *N* = 4 WT YD, *N* = 4 *Sgca* null YD mice and *N* = 3 *Sgca* null RD mice fed for 5 weeks. For the 25-week group, *N* = 4 *Sgca* WT YD, *N* = 3 *Sgca* null YD mice and *N* = 3 *Sgca* null RD mice. Results are means ± SD; Two-tailed unpaired Student’s *t*-test; *P < 0.05; **P < 0.01. **b** SDH on Tibialis Anterior muscle section *Sgca* null YD or RD mice fed for 5 or 25 weeks. Scale bar 500 μm. *N* = 6 *Sgca* null YD and 5 *Sgca* null RD mice fed for 5 weeks. For the 25 weeks, group *N* = 6 *Sgca* null YD and RD mice. **c** MyHC I positive fibers (green) and laminin (red) Immunofluorescence of Tibialis Anterior sections of *Sgca* null mice fed with YD or RD for 5 or 25 weeks. Scale bar 200 µm. *N* = 3 for *Sgca* null YD and RD mice in the 5-week group. *N* = 3 for *Sgca* null YD mice and *N* = 4 for *Sgca* null mice RD in the 25-week group. **d** Quantification of MyHC I positive fibers in Tibialis Anterior muscles of *Sgca* null mice fed with YD or RD for 5 or 25 weeks. *N* = 3 for *Sgca* null YD and RD mice in the 5-week group. *N* = 3 for *Sgca* null YD mice and *N* = 4 for *Sgca* null mice RD in the 25-week group. Results are means ± SD; Two-tailed unpaired Student’s *t*-test; **P < 0.01. **e** Ultrastructure of EDL of *Sgca* null mice fed with YD or RD. At low magnifications (scale bar 1 µm) the endoplasmic reticulum (narrow arrow), the Z line (thick arrow) and mitochondria (triangle) are underlined. At higher magnifications (scale bar 500 nm), degradation sites of thin filament (arrowhead) are visible. **f** Western Blot of VDAC expression in *Sgca* null mice YD or RD mice fed for 5 or 25 weeks. Vinculin was used to normalize. *N* = 3 for *Sgca* null YD mice and *N* = 3 for *Sgca* null RD mice in 5 and 25 groups.

To dissect the molecular dynamics modulated by cyanidin, we assessed its anti-oxidant ability by measuring the index of protein oxidation^25,26^, calculated as protein carbonylation content (PCC) from quadriceps protein extracts of both RD- and YD-fed *Sgca* null mice. As shown in Fig. 3a, YD-fed dystrophic animals progressively accumulate higher PCC compared to the RD-fed counterpart. Interestingly, the latter reached levels of PCC as low as the WT conditions. The remarkable drop of PCC in RD-fed animals might represent the synergistic outcome of increased oxidative metabolism and mitochondrial activity^27^. Based on this evidence, we performed succinate dehydrogenase (SDH) staining on TA sections from *Sgca* null mice following YD or RD supplementation. SDH staining is an enzymatic assay able to stain fibers with oxidative metabolism owing to their mitochondrial SDH activity. The outcomes confirmed an increased number of oxidative fibers in the TA of *Sgca* null mice fed with RD compared to their YD-fed littermates (Fig. 3b).

It has been described both in dystrophic murine models and in patients that muscle fibers expressing high levels of myosin heavy chain isoform I (MyHC I) are preserved from MD progression as a consequence of their stronger antioxidant capacity^24,28^. The immunostaining for MyHC I^+^ slow-twitching muscle fibers in the TA sections of RD-fed animals corroborates both the metabolic and the muscle fiber phenotype switch induced by this nutraceutical approach (Fig. 3c). The quantification indeed confirmed a significant shift towards an oxidative muscle metabolism following short- and long-term cyanidin supplementation (Fig. 3d). To follow-up on these findings, we parse the ultrastructure of *Extensor Digitorum longus* (EDL) muscle fibers in *Sgca* null mice fed with or without cyanidin for 12 weeks. Low magnification-electron microscopy analysis highlighted structural abnormalities in terms of sarcomere organization, mitochondria appearance, and distribution in YD-fed dystrophic mice (Fig. 3e and S1e). Conversely, RD-fed dystrophic mice presented with a healthier sarcomeric organization and increased mitochondrial amount, whereas only YD-fed dystrophic mice were characterized by aberrantly extended regions of endoplasmic reticulum, as a condition of sarcoplasmic stress. While disease progression causes flawed mitochondria and selective loss of the thin filaments, cyanidin supplementation was sufficient to improve mitochondrial number and morphology, encompassed by the preservation of sarcomere structure at thin filaments. Interestingly, RD-fed *Sgca* null animals show a thicker Z line as a key feature of MyHC I^+^ fibers^27^, confirming the data observed from the MyHC immunostaining. Consistent with electron microscopy, the mitochondrial mass, revealed by VDAC, was increased in muscles from 25-week-RD-fed mice (Fig. 3f and S1f).

### A cyanidin-enriched diet ameliorates the late-stage dystrophic phenotype

**Fig. 4:**
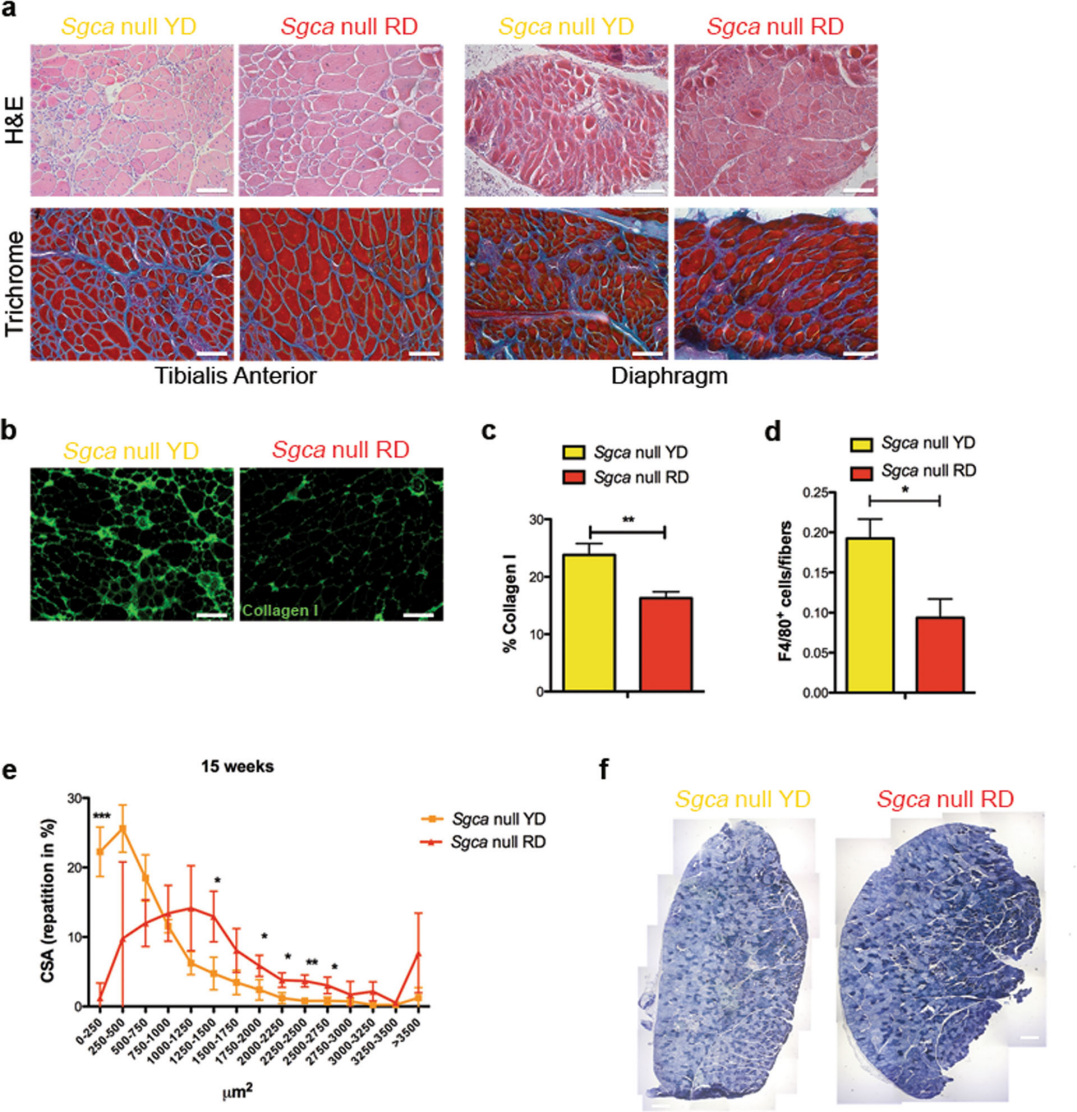
Cyanidin-enriched diet ameliorates dystrophic phenotype also in already compromised *Sgca*-null mice. **a** Hematoxylin and eosin (H&E) and Milligan’s Trichrome staining on Tibialis Anterior and Diaphragm muscles of 5-week-old *Sgca* null mice fed with YD or RD for 15 weeks. Scale bar 100 µm. *N* = 3 *Sgca* null YD mice and *N* = 3 *Sgca* null RD mice. **b** Collagen I Immunofluorescence (green) and nuclei (blue) in Tibialis Anterior sections of 5-week-old *Sgca* null mice fed with YD or RD for 15 weeks. Scale bar 100 μm. *N* = 4 *Sgca* null YD mice and *N* = 4 *Sgca* null RD mice. **c** Quantification of Collagen I^+^ positive area in Tibialis Anterior sections of 5-week-old mice fed with YD or RD for 15 weeks. *N* = 3 *Sgca* null YD and RD mice. Results are means ± SD; Two-tailed unpaired Student’s *t*-test; **P < 0.01. **d** Quantification of F4/80^+^ macrophages per myofibers in Tibialis Anterior sections of 5-week-old mice fed with YD or RD for 15 weeks. *N* = 3 *Sgca* null YD and RD mice. Results are means ± SD; Two-tailed unpaired Student’s *t*-test; *P < 0.05. **e** Distribution of myofibers Cross Sectional Area (CSA) of Tibialis Anterior of 5-week-old *Sgca* null mice fed with YD or RD for 15 weeks. *N* = 3 for *Sgca* null YD and RD mice. Results are means ± SD of at least three independent experiments; Two-tailed unpaired Student’s *t*-test; *P < 0.05. **f** SDH staining on Tibialis Anterior muscle section of 5-week-old *Sgca* null mice fed with YD or RD for 15 weeks. Scale bar 100 μm. *N* = 3 for *Sgca* null YD and RD mice. Results are means ± SD; Anova test was used. **P < 0.01; ***P < 0.001.

Sclerosis and inflammatory infiltrates characterize advanced stages of MD and hamper the prospective successful outcome of cell and gene delivery protocols, precluding the treatment for a large portion of dystrophic patients. In a therapeutic framework, we tested whether cyanidin offers remarkable benefits even when supplied at later stages of the disease, as this situation more likely represents the realistic clinical setting. In this protocol, the RD or the cyanidin-mock equivalent YD was supplied for 5 weeks to 15-weeks-old *Sgca* null mice (Fig. S1d), yet when early signs of dystrophy arise^22^. Hematoxylin and Eosin staining on TA and diaphragm muscles of RD-fed *Sgca* null mice revealed remarkable amelioration of muscle organization and morphology that appears more preserved and with less cell infiltrates (Fig. 4a, upper panels). Trichrome staining on TA and diaphragm sections also showed a decrease in extracellular matrix deposits when RD is supplied, at variance with *Sgca* null mice fed with the control diet (Fig. 4a, bottom panels). The diet-induced benefit on fibrotic degeneration was further consolidated by quantifying the fluorescent positive area of TA sections stained for collagen I (Fig. 4b), to reveal a considerable drop of its deposition in RD- vs. YD-fed dystrophic mice (Fig. 4c).

To parse the inflammatory milieu, we quantified the number of F4/80^+^ macrophages in TA sections from YD- versus RD-fed *Sgca* null mice, revealing less macrophage infiltration in *Sgca* null mice upon cyanidin supplementation (Fig. 4d).

The homogenous fiber calibre distribution observed in RD- vs YD-fed dystrophic mice corroborates our previous results (Fig. 4e). Similarly to what has been observed at weaning (Fig. 3b), *Sgca* null animals fed with the RD at 5-weeks old shifted towards an oxidative fiber metabolism as from the SDH activity assay (Fig. 4f).

## Discussion

MDs represent a heterogeneous group of inherited pathologies, some are life-threatening conditions while others are still lacking a definitive therapy. The pathological hallmark consists of muscle degeneration loops followed by unsuccessful regeneration attempts to repair the damaged tissue. As a net outcome of this iteration, SC pool’s exhaustion initiates the progressive replacement of muscle by connective and adipose tissue, which compromise functionality and lead to the loss of ambulation at childhood and ultimately to premature death.

It is widely acknowledged that mitochondrial dysfunction and cellular energy perturbations serve as complementary mechanisms hastening muscle degradation. Indeed, dystrophic muscle is associated with a decrease in muscle mitochondrial content^29,30^. Previous studies reported the dystrophic muscle fibers to be intrinsically susceptible to oxidative stress, which emerges as the ground state underpinning pathology progression^7,31^. However, the mechanistic dynamics governing this phenomenon within the muscle milieu have been poorly characterized.

Proper nutrition, assumed as the optimal intake of bioactive compounds might be at the basis of innovative adjuvant therapies in patients with states of chronic muscle wasting^32^. As such, nutraceuticals aimed to target the metabolic reprogramming, are paving the way for auxiliary protocols in the treatment of MDs. For instance, specific aminoacidic formulation facilitates blood and oxygen supplies to the female *mdx* mice thus mitigating disease progression^33^.

Importantly, the expectations about the potential impact of nutraceutical options in the treatment of MDs must be realistic and far to presume they could reverse very advanced stages such as loss of ambulation. However, the implementation of these treatments could act as a disease modifier that hinders the pathological course, having beneficial effects on patients’ healthspan and increasing their eligibility for curative options.

In this study, we show that a cyanidin-enriched diet delay the pathological onset and improve muscle performance in a pre-clinical model of MD. Among the wide portfolio of animal models, we focused on the *Sgca* null dystrophic mouse, since it presents with severe degenerative myopathy very similar to Duchenne human patients since the early life stage^22^. We demonstrated the nutritional benefits of cyanidin supplementation in the disease progression of *Sgca* null mice, inducing important ameliorations both in terms of tissue morphology and muscle performance.

State-of-the-art knowledge recognizes slow-twitching fibers, with a prevalent oxidative metabolism, more prone to resist MD degeneration when compared to the glycolytic fast-twitching fibers^20^. Among others, our previous studies remarked the relevance for an oxidative fiber-type metabolism together with the expression of MyHC I isoform in both preserving muscular architecture and delaying the signs of the dystrophic progression^24,28,34^. Here, dietary supplementation with cyanidin promotes a metabolic shift towards oxidative muscle fibers and enhanced mitochondrial biogenesis, and ultimately, shield against the pathology progression.

Macrophages act as a cellular cornerstone playing both pro- and anti-inflammatory roles during muscle recovery. Chronic muscle injury, a key feature of dystrophic setting, provokes macrophage infiltrations which participate in the worsening of the disease and preventing macrophage infiltration improves dystrophic muscle^6,35^. We observed a remarkable reduction of macrophage infiltration when *Sgca* null mice were assigned to a diet supplemented with a cyanidin-rich preparation.

The safety and toxicological concerns about anthocyanins consumption in humans are remarkably low. There are no reports about adverse health effects associated with the consumption of anthocyanins at the usual dietary intake levels. Although most anthocyanins have reduced bioavailability, the cyanidin-3-glucoside (used in our study) ranks among the more stable with a relatively low-dose threshold to produce its protective results against oxidative stress both in vitro and in vivo^36,37^.

The health-caring effect of anthocyanins has been successfully proven against cardiovascular diseases, where 5-week-regimen with a purple corn diet was sufficient to protect from ischemic injury or to prevent the cardiotoxic effects of doxorubicin (Dox), a chemotherapeutic agent used for the treatment of breast cancer^20,21^. Currently, trial intervention in breast cancer-bearing patients undergoing radiotherapy is testing the beneficial effect of the administration of a product enriched in anthocyanins on the inflammatory response to radiation and on its consequent skin toxicity, as well as on systemic low-grade inflammation reaction^38^.

Our findings leverage the nutritional benefits of cyanidin in a model of chronic muscle condition where oxidative stress and inflammation trigger progressive tissue wasting and drive the detrimental outcomes of the pathology. In a dystrophic context, we provide compelling evidence that either short- or long-term supplementation of cyanidin, administered when the disease is either still pre-symptomatic (at weaning) or advanced, preserves muscle functionality thanks to its bivalent nature of anti-oxidant and anti-inflammatory agent. To our knowledge, this feature renders cyanidin unique among other dietary antioxidants, such as Resveratrol or Sulphorophane^6,13^. The inflammatory healing effects of anthocyanins have been previously observed in models of systemic inflammation, such as hepatic swelling, chronic pain conditions and recently obesity-associated inflammation^39,40^.

The modulation of fiber-type specificity coupled with a balance towards slow-twitch oxidative fibers provides a synergic protective mechanism prompted by the cyanidin-enriched diet, triggering the endogenous anti-oxidant response.

The biological significance of our findings entails extensive therapeutic implication since it reveals how nutritional-based intervention, intended to mitigate the cross-talk between oxidative stress and inflammatory cues, hamper the progression of MD.

The current study identifies cyanidin as a first-in-class natural compound that alleviates the progression of a degenerative genetic disease upon dietary consumption, representing an adjuvant nutritional-based intervention for MDs. Based on its documented potential to hinder dystrophic signs even at the advanced stage, when patients become ineligible for primary interventions such as gene- or cell-based protocols, cyanidin dietary supplementation may restore muscle conditions amenable to definitive therapeutic treatments, raising the likelihood of successful outcomes.

While experimental interventions aimed to correct the genetic cause of MDs still represent the mainstay therapeutic option, our findings advocate that nutraceutical supplementation with cyanidin holds promise as an auxiliary strategy in humans to target the multiple metabolic abnormalities of MDs and maintain functional muscle mass, further warranting successful clinical perspectives to an extended cohort of dystrophic patients.

## Methods

### Maize production

Maize genotypes were originally in W22 background, homozygous dominant for the *a1*, *a2*, *c1*, *c2*, *bz1* and *bz2* genes, homozygous recessive for the *r1* gene and different *b1 pl1* constitution. To obtain cyanidin-rich and cyanidin-free corn with an isogenic background, a maize cyanidin-rich hybrid was used carrying the *B1* and *Pl1* alleles (Red diet, RD), which confer purple pigmentation in seed pericarp and all plant tissues^41–44^. Plant and seed tissues carrying *b1 pl1* alleles are cyanidin-free (Yellow diet, YD). To obtain ears with a high production of kernels, the homozygous inbred line *B1 Pl1* W22 and the *b1 pl1* W22 inbred line were crossed to a *b1 pl1* B73 inbred line and the F1 progeny seeds were used to produce two synthetic populations differing only in *b1 pl1* constitution^21^.

### Mouse model

All mice were kept in pathogen-free conditions with 12–12 h light-dark cycle. All the procedures on animals were conformed to Italian law (D. Lgs n 2014/26, implementation of the 2010/63/UE) and approved by the University of Milan Animal Welfare Body and by the Italian Ministry of Health. The genotyping strategies have been published in the animal reference. *Sgca* null mice were previously described in Duclos et al.^22^. At 3 weeks of age, *Sgca* null mice were randomly divided into two groups: one fed with the control cyanidin-free diet (YD) and the other one fed with the cyanidin-enriched diet (RD). Diets were supplied ad libitum for 5 or 25 weeks. Both male and female mice were used indiscriminately. To check the effects of the cyanidin-enriched diet on mice in adulthood, we also supplied the diets in *Sgca* null animals at 5 weeks of age for 15 weeks.

### Haematoxylin and eosin and Milligan’s trichrome

Haematoxylin and eosin staining was performed on 7 μm-thick cryosections fixed with 4% paraformaldehyde for 10 min at 4 °C. The staining was performed according to standard protocols. For Milligan’s trichrome staining, sections were fixed for 1 h with Bouin’s fixative (Sigma-Aldrich) and rinsed for 1 h under running water. Sections were then rapidly dehydrated to 95% EtOH in graded ethanol solutions, successively passed in 3% potassium dichromate (Sigma-Aldrich) for 5 min, rapidly washed in distilled water, stained with 0,1% acid fuchsin (Sigma-Aldrich) for 30 s, washed again in distilled water, passed in 1% phosphomolybdic acid (Sigma-Aldrich) for 3 min, stained with Orange G (2% in 1% phosphomolybdic acid) (Sigma-Aldrich) for 5 min, rinsed in distilled water, passed in 1% acetic acid (VWR) for 2 min, stained with 1% Fast Green for 5 min (Sigma-Aldrich), passed in 1% acetic acid for 3 min, rapidly dehydrated to 100% EtOH and passed in Xylene before mounting with Eukitt (Bio-Optica).

### SDH staining

For SDH staining, freshly cut 7μm-thick cryosections of *Tibialis anterior* were used. Sections were incubated in SDH incubating solution (1 tablet of nitroblue tetrazolium dissolved in 0.1 M sodium succinate-0.1 M phosphate buffer pH7.4, all from Sigma-Aldrich) for 1 h at 37 °C, rinsed in distilled water, rapidly passed in 30%, 60%, 30% Acetone (VWR), and rinsed again in distilled water. Sections were then rapidly dehydrated in graded EtOH solutions, cleared in Xylene and mounted with Eukitt mounting medium.

### Treadmill test

For Treadmill test functional assay, 3-week-old *Sgca* null mice were fed for 5 weeks with RD or YD diet and WT with YD diet, as control. Mice were trained three times once a week before recording the performance. Treadmill test was therefore performed starting from 8-week-old mice, once a week for 15 weeks. The test was conducted on a treadmill (Bioseb) with a 10% incline, starting from a speed of 6 cm/s and increasing it by 2 cm/s every 2 min. For each test, the time to exhaustion of each mouse was measured.

### Protein extraction and Western Blot

Western blot was performed on protein extracts from muscles homogenized in Tissue Buffer (150 mM Tris-HCl, pH 7.5; 1 mM EDTA, 1% Triton, 150 mM NaCl, all from Sigma-Aldrich) for 30 s, followed by lysis on ice for 30 min and by centrifugation at 10000 rpm at 4 °C to pellet cell debris. The supernatant was quantified using DC Protein Assay (Biorad), and 30–50 μg of total protein extracts were loaded for each sample. Images were acquired using Chemidoc ImageLab software (Biorad). The following antibodies and dilutions were used: rabbit anti-VDAC1 (1:10,000, Cell Signaling), mouse anti-Vinculin (1:2500, Sigma-Aldrich).

### Protein carbonylation content (PCC)

The level of oxidative stress in quadriceps protein extracts was quantified by measuring protein carbonylation. Carbonyls groups were derivatized into their DNP adducts using 2,4-Dinitrophenylhydrazine (DNP)^25^. Quadriceps muscles were homogenized in tissue buffer adding 1 mM DTT. After protein quantification 50 μg of protein was derivatized with the same volume of DNPH (10 mM in 2 M HCl, Sigma-Aldrich) for 1 h in the dark at RT. Afterward, to precipitate carbonylated protein and stop the derivatization reaction a solution 30% of trichloroacetic acid (TCA, Sigma-Aldrich) was added and sample incubated for 15 min on ice. Then, samples were centrifuged at 15,000 × *g* for 15 min at 4 °C. After removing the supernatant, each pellet was washed three times with a solution of ethanol-ethyl acetate (1:1) to remove the excess of DNPH, then the pellet was solubilized in 1 mL of guanidine (6 M, Sigma-Aldrich) and incubated for 30 min at 37 °C. The content of carbonylated protein was measured by a spectrophotometer at 370 nm (Janway).

### Immunofluorescence

Immunofluorescence was performed on 7-μm cryosections. Slices were fixed for 10 min at 4 °C with 4%PFA, washed twice in PBS and permeabilized with a solution containing 1%BSA (Sigma-Aldrich) and 0.2% Triton X-100 (Sigma-Aldrich) in PBS, for 30 min at room temperature. After blocking for 30 min with 10% donkey serum, slices were incubated O/N with primary antibodies in PBS-1.5% donkey serum. The day after, two washes in PBS-1%BSA-0.2%Triton X-100 were performed and samples were incubated for 45 min at room temperature with secondary antibodies and Hoechst (1:500, Sigma-Aldrich). The excess of antibody was washed twice in PBS-0.2%Triton X-100 before mounting with Fluorescence Mounting Medium (Dako). The following antibodies and dilutions were used: goat anti-Collagen I (1:200, Southern Biotech), donkey anti-goat 488 (1:250, Jackson Lab).

F4/80 immunofluorescence was performed on cryosections of Tibialis Anterior. Samples were permeabilized for 10 min at RT in a solution of PBS-0.5% Triton X-100 (Sigma-Aldrich), then washed three times in PBS. After blocking for 30 min with a solution of PBS-3%BSA (Sigma-Aldrich), slices were washed twice with PBS and incubated O/N with primary antibodies anti-F4/80 (rat, 1:400, Novus) and anti-laminin (rabbit, 1:300 Sigma-Aldrich) in PBS. The day after, three washes in PBS were performed and samples were incubated for 45 min at room temperature with secondary antibodies and Hoechst (1:500, Sigma-Aldrich). The excess of antibody was washed five times in PBS before mounting with Fluorescence Mounting Medium (Dako). F4/80 positive cells were normalized on the total number of fibers in the picture.

For slow Myosin heavy chain (MyHC I) immunostaining, cryosections were retrieved in a Na-citrate solution (10 mM pH 6) for 30 min in a steamer machine. Once equilibrated the section at RT, slices were washed twice with PBS for 10 min and then incubated with the primary antibody mouse anti-MyHC I 1:1000 (Sigma-Aldrich) and rabbit anti-laminin 1:300 (Sigma-Aldrich) O/N at 4 °C. Fibers positive for MyHC I were normalized on the total number of myofibers.

### Transmission electron microscopy (TEM)

For TEM, EDL muscles were dissected from sacrificed animals, pinned on a Sylgard dish, fixed at room temperature with 3.5% glutaraldehyde in 0.1 M NaCaCO buffer (pH 7.4), and stored in the fixative at 4 °C. Fixed muscles were then post-fixed in a mixture of 2% OsO_4_ and 0.8% K_3_Fe(CN)_6_ for 1–2 h, rinsed with 0.1 M sodium cacodylate buffer with 75 mM CaCl_2,_ en-block stained with saturated uranyl acetate, and embedded for EM in epoxy resin (Epon 812) as in ref. ^45^. Ultrathin sections (~40 nm) were cut in a Leica Ultracut R microtome (Leica Microsystem, Austria) using a Diatome diamond knife (DiatomeLtd. CH-2501 Biel, Switzerland) and examined at 60 kV after double- with uranyl acetate and lead citrate, with a FP 505 Morgagni Series 268D electron microscope (FEI Company, Brno, Czech Republic), equipped with Megaview III digital camera (Munster, Germany) and Soft Imaging System (Germany).

### Macrophages isolation from skeletal muscle

Skeletal Muscles collected from hind limb were minced and digested enzymatically and mechanically in a single cell suspension with 0.2% of Collagenase B (Roche) in RPMI medium (Lonza) for 1 h and 30 min in a water bath at 37 °C under agitation. After filtration with cell strainers (70 and 40 µm, Grainer) and centrifugation at 272 × *g* for 10 min at 4 °C, the single cell suspension was resuspended in sterile PBS-0.5% BSA (Genespin)-2 mM EDTA (Sigma-Aldrich) and incubated with anti-CD45 antibody conjugated with magnetic beads (Miltenyi Biotech) for 30 min at 4 °C. Cells were then washed with PBS-0.5% BSA (Genespin)-2 mM EDTA (Sigma-Aldrich) and CD45^+^ cell isolation was performed by using magnetic columns (Miltenyi, Biotech) according to manufacturer instructions. After Fc blocking (Fc Buffer, Miltenyi Biotech), the fraction of CD45^+^ cells was incubated with Ly6C-PE antibody (eBioscence), to discriminate pro-inflammatory (Ly6C^+^) from anti-inflammatory macrophages (Ly6C^−^), and with CD64-APC antibody (BD Bioscience) to discriminate neutrophils from macrophages. Cell sorting experiments were then performed using a FACSAria II (BD Bioscience). Diva software (BD Pharmingen, San Diego, CA) was used for data acquisition and analysis.

### Image acquisition

Images were acquired with an inverted microscope (Leica-DMI6000B) equipped with Leica DFC365FX and DFC400 cameras. The Leica Application Suite software was used for acquisition while Photoshop was used to generate merged images.

### Measurement of myofiber CSA and Collagen I quantification

Measurement myofiber CSA was performed on Tibialis Anterior muscle sections using Image J software. Collagen I quantification was performed using a Macro in ImageJ to identify and quantify Collagen I positive areas.

### Statistics

All data shown in the graphs are expressed as mean ± SD, apart from graphs showing CSA distributions, which are expressed as mean ± whiskers from min to max. Statistical analysis between two columns was performed using two-tailed unpaired Student’s *t*-test, whereas data containing more than two experimental groups were analyzed with one-way ANOVA followed by Bonferroni’s test. *P < 0.05; **P < 0.01; ***P < 0.001; confidence intervals 95%, alpha level 0.05.

## Supplementary information

Supplementary Figure S1

Supplementary Figure S1 Legend
