# Supplementary figures and images for: Nutritional intervention with cyanidin hinders the progression of muscular dystrophy
Source: Cell Death Dis. 2020 Feb 18;11(2):127. doi: 10.1038/s41419-020-2332-4 (PMC7028923; doi:10.1038/s41419-020-2332-4)

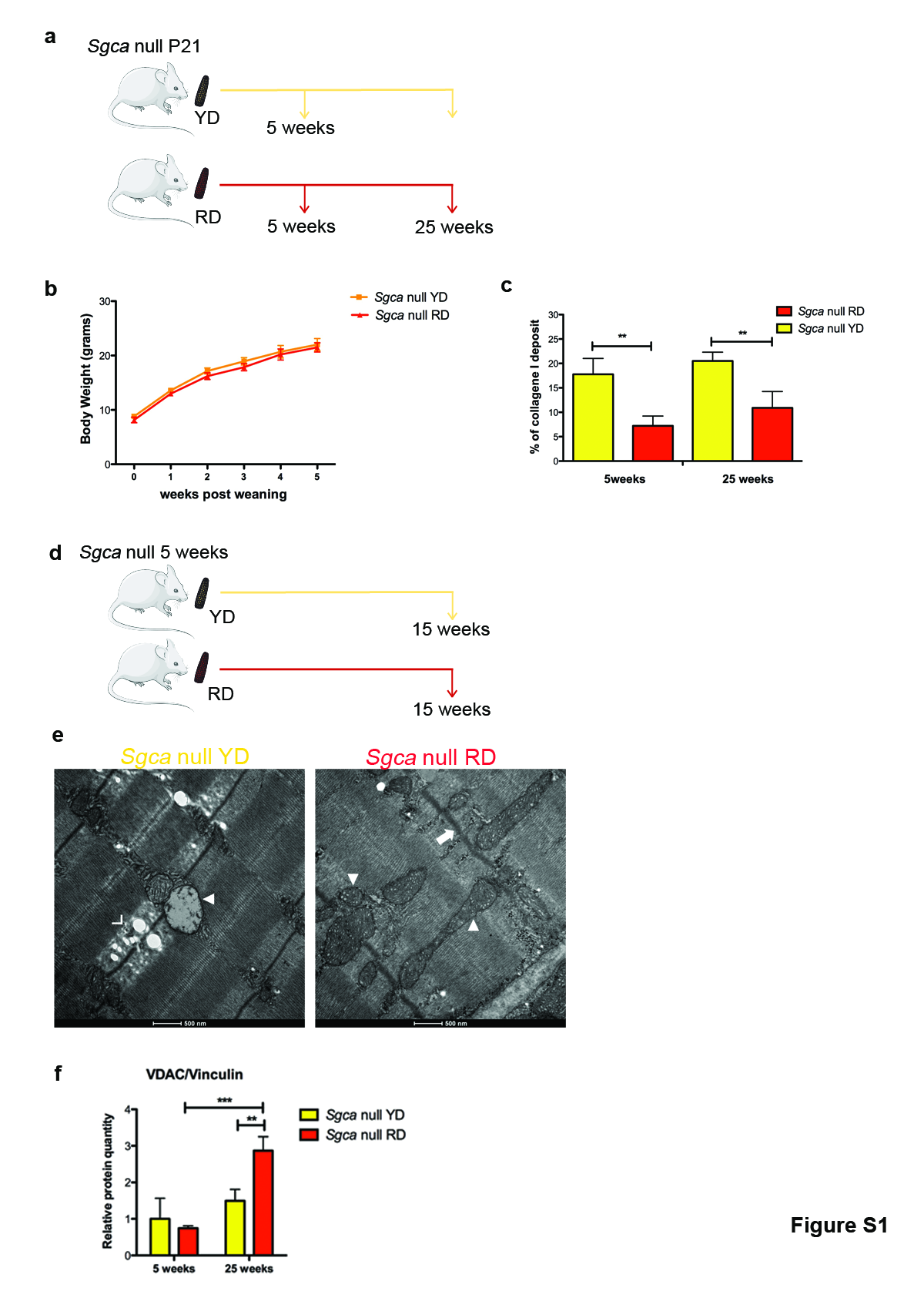

Supplement: Supplementary file 1 — Supplementary Figure S1 [file 41419_2020_2332_MOESM1_ESM.tif]
