# Supplementary material for: Nutritional intervention with cyanidin hinders the progression of muscular dystrophy
Source: Cell Death Dis. 2020 Feb 18;11(2):127. doi: 10.1038/s41419-020-2332-4 (PMC7028923; doi:10.1038/s41419-020-2332-4)
Supplement: Supplementary file 2 — Supplementary Figure S1 Legend [file 41419_2020_2332_MOESM2_ESM.docx]

**Supplementary Figure 1**

a) Scheme of the first nutritional protocol. At weaning (P21), *Sgca* null mice are fed with YD or RD for 5 or 25 weeks. b) Weight of *Sgca* null mice fed with YD or RD from weaning to 2 months of age. N=4 *Sgca* null YD and RD mice. Results are means ± SD; Two-tailed unpaired Student’s t-Test. c) Quantification of Collagen I^+^ positive area in Tibialis Anterior sections of *Sgca* null mice fed with YD or RD for 5 or 25 weeks. Results are means ± SD; Two-tailed unpaired Student’s t-Test; **P<0,01. d) Scheme of the second nutritional protocol. At 5 weeks of age, *Sgca* null mice fed with YD or RD for 15 weeks. e) Ultrastructure of EDL of *Sgca* null mice fed with YD or RD. The Z line (thick arrow) and mitochondria (triangle) are underlined. Degradation sites of thin filament (arrow head) are visible in the EDL of *Sgca* null mice fed with YD (scale bar 500nm). f) Quantification of the VDAC Western Blot. Vinculin was used to normalize. N=3 for *Sgca* null YD mice and N=3 for *Sgca* null RD mice in 5 weeks and 25 groups.
